# Relapsed or refractory central nervous system lymphoma successfully treated by glofitamab combined with lenalidomide

**DOI:** 10.3389/fonc.2025.1685271

**Published:** 2025-11-03

**Authors:** Yin-yin Peng, Xiao-qiong Tang

**Affiliations:** Department of Hematology Medicine, The First Affiliated Hospital of Chongqing Medical University, Chongqing, China

**Keywords:** central nervous system lymphoma, primary, secondary, diffuse large B-cell lymphoma, relapsed, refractory

## Abstract

**Background:**

Central nervous system lymphoma (CNSL) is rare and aggressive, which has high rates of recurrence and fatality. At present, there does not exist any standard treatment for the relapsed/refractory (R/R) CNSL.

**Methods:**

We retrospectively analyzed 4 patients with R/R CNSL, who were treated with gloftamab combined with lenalidomide between October 2024 and June 2025 at the First Affiliated Hospital of Chongqing Medical University. Treatment response was assessed by brain magnetic resonance imaging and cerebrospinal fluid cytology every two cycles.

**Results:**

The 4 R/R CNSL patients include 1 male and 3 females, with the mean age of 58 years (range: 51~69 years). Their clinical manifestations at relapse included headache, stupor, listlessness, lethargy, nausea, poor appetite, weak limbs, walking disorder, slurred speech and visual impairment. The mean relapse time was 5 months (range 3~8 months) after the last treatment. After 2 cycles of treatment, all patients achieved rapid remission (2 in CRu and 2 in PR), the overall response rate was 100% (4/4). They got deep remission after 4 cycles (3 in CR/CRu, 1 in PR). No patient experienced cytokine release syndrome, immune effector cell-associated neurotoxicity syndrome nor hematological toxicity of grade 3 or above. Neither liver nor kidney dysfunction was observed. No treatment discontinuation occurred due to adverse events.

**Conclusions:**

The glofitamab-lenalidomide combination showed promising activity and excellent tolerability in R/R CNSL, potentially addressing the critical need for effective salvage regimens.

## 1 Introduction

Central nervous system lymphoma (CNSL) is a rare and aggressive subtype of non-Hodgkin’s lymphoma (NHL). It can be either primary central nervous system lymphoma (PCNSL) or secondary central nervous system lymphoma (SCNSL), depending on whether there is a systemic involvement (1).

PCNSL often involves the brain parenchyma, meninges, spinal cord, or eyes, in the absence of other systemic infiltration at the time of initial diagnosis (2). PCNSL is rare, which accounts for 4~6% of all extranodal NHL and 4% of all newly diagnosed central nervous system (CNS) malignancies (3). According to the 5^th^ edition of the World Health Organization (WHO) classification for haematolymphoid tumous: lymphoid neoplasms, PCNSL is a distinct subtype of aggressive lymphoma that belongs to lymphoma of immune-privileged site (4). Histopathology classifies more than 90% of PCNSL as diffuse large B-cell lymphoma (DLBCL) (5). Gene expression profiling methods recognize the majority of PCNSL cases as the activated B-cell-like (ABC) or non-germinal center B-cell-like (non-GCB) subtype (6). As PCNSL is aggressive, its prognosis is poor. If untreated, patients with PCNSL usually die within 1~3 months (7). Despite the efficacy of methotrexate (MTX) -based therapy, 50% of PCNSL cases may relapse in the next 10 to 18 months, and 10% to 15% of PCNSL cases may prove to be refractory. Prognosis for the relapsed/refractory (R/R) PCNSL is poor, with an estimated overall survival (OS) of 2 months for patients with refractory disease and 3.7 months for those with relapsed disease (8).

SCNSL is defined as the aggressive lymphoma involved in the CNS, either at the time of initial diagnosis of systemic lymphoma or in the setting of relapse. It can be either isolated or with synchronous systemic disease (9). The incidence and timing for SCNSL vary based on histology. DLBCL accounts for the majority of cases across aggressive histologies (10). The median time from first pathologic diagnosis of DLBCL to SCNSL was 9 months, and 20% patients developed SCNSL during first-line immunochemotherapy. The outcome of SCNSL is poor, with 2-year OS <20% (11).

DLBCL is the main pathological type of PCNSL and SCNSL. The treatment of SCNSL caused by DLBCL infiltration of CNS mainly refers to PCNSL-DLBCL. Therefore, here we only focus on the DLBCL type of CNSL, and the CNSL contents hereafter only refer to the DLBCL type of CNSL. Although high-dose MTX-based chemotherapy is considered to be an effective treatment for CNSL, its long-term survival rate is still very low. For R/R patients, the subsequent treatment should be selected according to the age, performance status, initial treatment plan, recurrence time, and the will of patients. At present, there is not any standard treatment for the R/R CNSL.

High-dose chemotherapy regimens and autologous stem cell transplantation (ASCT) consolidation can be used as salvage therapies in R/R CNSL if not administered as first-line treatment (12). However, the prognosis of R/R CNSL is still poor, with a median survival of approximately 7 months, dropping to 2 months without treatment (13). Although guidelines recommend whole-brain radiotherapy (WBRT) or ASCT, their usage is limited due to the late neurotoxicity which is associated with radiotherapy, as well as the high mortality of myeloablative chemotherapy prior to ASCT.

Multiple novel molecular targeted drugs and chimeric antigen receptor T-cell (CAR-T) therapy are with ongoing clinical trials for R/R CNSL patients (14). CAR-T therapy have been used in PCNSL in several small-scale studies, however, only a specific population can benefit and have more durable responses, besides, unaffordable price, severe cytokine-release syndrome (CRS) and immune effector cell-associated neurotoxicity syndrome (ICANS) limit its use. Bruton tyrosine kinase inhibitor (BTKi) (15), programmed cell death protein 1 (PD-1) inhibitor (16), phosphoinositide 3-kinase (PI3K) inhibitor (17), exportin 1 (XPO-1) inhibitor (18), immunomodulatory drug (19–22) have proven to be effective methods in CNSL patients, but most of relevant clinic studies have only small samples. Moreover, although it’s shown single agent therapy is effective, a combination therapy is often expected to have larger potential benifits than only single-agent therapy. Therefore in clinic, combinations of targeted therapy and immunochemotherapy are of great interests and may play important roles in this situation (21). Glofitamab, a CD20×CD3 antibody, anther method of T-cell engager therapy except CAR-T, is considered to be one of the most promising treatment strategies in DLBCL (23). Patients receiving glofitamab therapy have lower incidence of CRS and ICANS than those receiving CAR-T therapy (24, 25). Lenalidomide is one of the immunomodulatory drugs. Previous studies (19–22) have shown that lenalidomide alone, or in combination with other drugs, is active in patients with R/R CNSL. It have been shown that lenalidomide can affect T-cell’s function by promoting immune synapse formation and stimulating the cytotoxic CD8+ and helper CD4+ T-cells (26). So lenalidomide combined with glofitamab may have synergistic effects on the CNSL. Here, we report 4 R/R CNSL patients treated with CD20×CD3 bispecific antibody glofitamab combined with lenalidomide therapy, in order to evaluate the efficacy and safety of glofitamab combined with lenalidomide therapy to these CNSL patients.

## 2 Materials and methods

In this study, we retrospectively analyzed 4 patients with R/R CNSL treated with gloftamab combined with lenalidomide between October 2024 and June 2025 at the First Affiliated Hospital of Chongqing Medical University. The study was approved by the ethics committee of the First Affliated Hospital of Chongqing Medical University (Number: 2025-383-01), and all patients have provided written informed consent in accordance with the Declaration of Helsinki.

The 4 patients all received craniotomy for brain tumor at first, and their tumor biopsy all showed positive of the CD20 and CD79 for CNS DLBCL, including 3 PCNSL patients and 1 SCNSL patient (**Table 1**). Two of them experienced relapse twice and received ASCT, while the other two experienced only once and were ineligible for ASCT either because of their poor physical conditions with Eastern Cooperative Oncology Group (ECOG) performance status ≥ 3, or because of their refusals to ASCT and WBRT. All patients were treated with the glofitamab combined with lenalidomide for a 21-day cycle. Pretreatment with obinutuzumab (1000mg) was administered intravenously 7 days before the first dose of glofitamab. Glofitamab was then administered intravenously with step-up dosing during cycle 1 (day 8: 2.5mg; day 15: 10mg), followed by fixed dose during cycles 2~12 (day 1: 30mg). To mitigate the risk of CRS, patients received dexamethasone 20mg intravenously, acetaminophen 1000mg orally, and isopropanazine 25mg intramuscularly, 1 hour before glofitamab therapy. Lenalidomide was administered orally with dose of 15mg/day continuously. Besides, aspirin was administered 100mg/day to all patients for prophylaxis of venous thromboembolism. If myelosuppression occurred, granulocyte colony-stimulating factor (G-CSF) or recombinant human thrombopoietin (rhTPO) would be administered.

**Table 1:** Clinical characteristics of the 4 R/R CNSL patients.

	No. 1 patient	No. 2 patient	No. 3 patient	No. 4 patient
Sex	male	female	female	female
Age (years)	54	58	69	51
Diagnosis	PCNSL, non-GCB,IELSG score 2, moderate-risk	PCNSL, non-GCB, IELSG score 3, moderate risk	PCNSL, non-GCB, IELSG score 3, moderate risk	SCNSL, DLBCL, non-GCB, IVA, IPI score 2, low-intermediate risk
Onset and Relapse	onset: left paraventricular basal ganglia. first relapse: left lateral ventricle, the para-temporal horn and the left temporal pole. second relapse: lateral ventricle, including the anterior horn and temporal horn of left lateral ventricle.	onset: right frontal lobe, corpus callosum, insular lobe, and partly in the right medial splenius capitis lymph node and bone marrow. relapse: right posterior horn of the ventricle and the left cerebellum.	onset: left frontal lobe and basal ganglia. relapse: right lateral ventricle of the centrum semiovale.	onset: breast. first relapse: right frontal lobe. second relapse: bilateral parieto-occipital lobe and the left splenium of corpus callosum.
Prior Therapies	RTM+BTKi (6 cycles), R+BTKi (2 cycles). RTCS (1 cycle), WBRT, ASCT	RTM+BTKi (4 cycles), BTKi	RTM+BTKi (4 cycles)	R-CHOP+MTX, radiotherapy, ASCT. RTM+BTKi (5 cycles), ASCT
Relapse Time	2024-12	2024-10	2024-12	2025-03
Time From The Last Therapy to Relapse	3 months	6 months	8 months	3 months
Previous Lines of Therapy	second line	first line	first line	second line
Symptoms at Relapse	weak limbs, walking disorder	nausea, poor appetite, listlessness, lethargy	slurred speech, stupor, poor appetite, walking disorder	headache, visual impairment
Therapy After Relapse				
No. of Glofitamab Cycles	8+	10+	8+	4+
Response after Therapy	PR	CR	CRu	CR
Adverse Event	grade 1 CRS and ICANS	grade 1 CRS and ICANS, grade 2 myelosuppression	grade 1 CRS	grade 2 CRS and ICANS, grade 1 myelosuppression

Treatment response was assessed by brain magnetic resonance imaging (MRI) and cerebrospinal fluid (CSF) cytology every two cycles, according to the criteria including complete response (CR), unconfirmed complete response (CRu), partial response (PR), stable disease (SD), and progressive disease (PD), which were suggested by the International Primary Central Nervous System Lymphoma Collaborative Group in 2005 (27). Treatment toxicities were evaluated according to the Patient-Reported Outcomes version of the Common Terminology Criteria for Adverse Events (PRO-CTCAE) of the National Cancer Institute (28). CRS and ICANS assessments and grading were conducted repeatedly according to the American Society for Transplantation and Cellular Therapy (ASTCT) CAR-T therapy toxicity criteria (29).

## 3 Results

The clinical characteristics of the 4 R/R CNSL patients are listed in **Table 1**, including 1 male and 3 females, with the mean age of 58 years (range: 51~69 years). Their clinical manifestations included headache, stupor, listlessness, lethargy, nausea, poor appetite, weak limbs, walking disorder, slurred speech and visual impairment. The mean relapse time was 5 months (range 3~8 months) after the last treatment. The MYD88 mutation was detected in the 4 patients by next generation sequencing of tumor tissue genes. No lymphoma cells were found in the the 4 CNSL patient’s CSF by flow cytometry analysis all the time.

**Figure 1:**
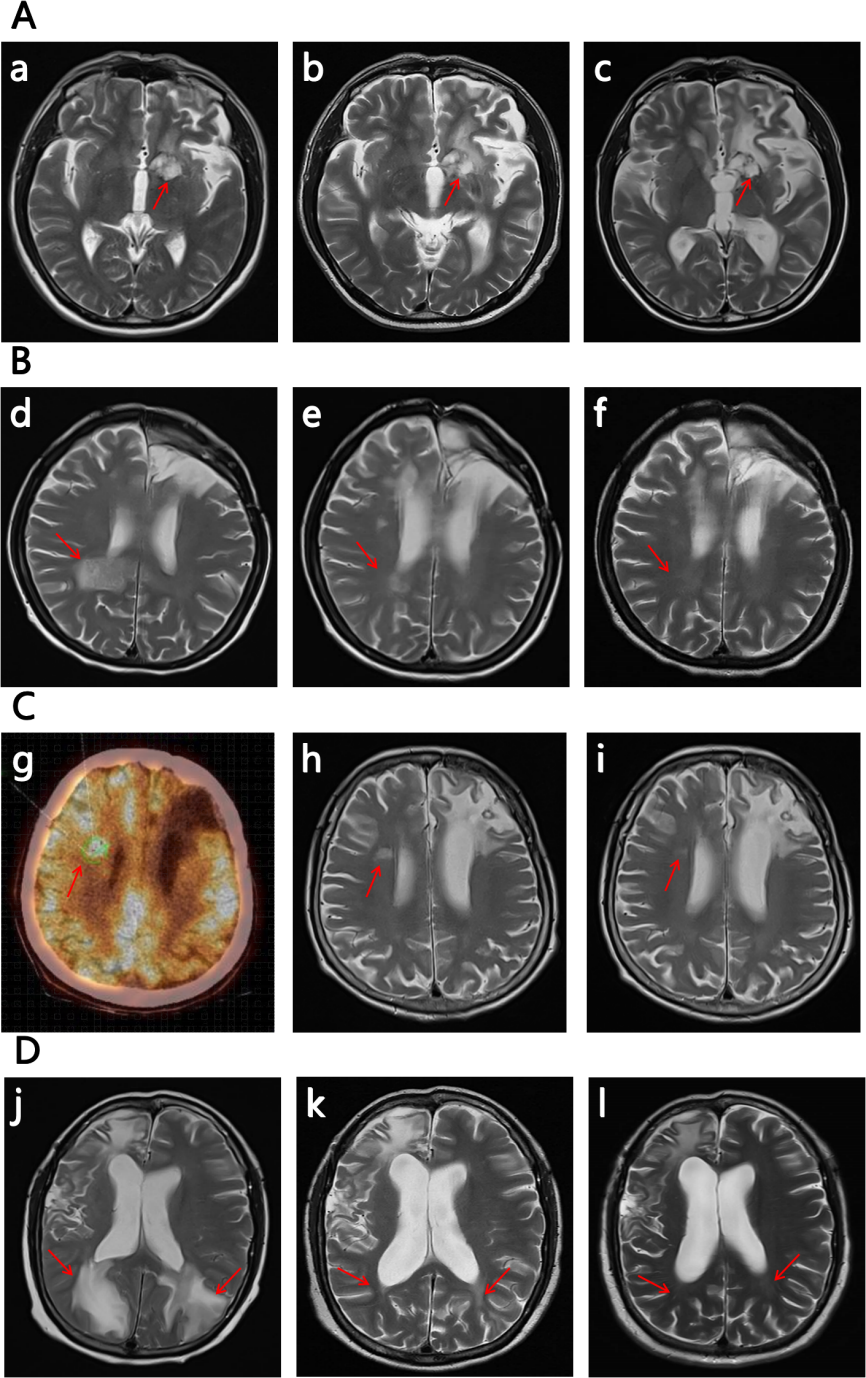
The images of the 4 R/R CNSL patient (the subfigure g was by PET-CT, while others were all by MRI). The significant decrease in the size and contrast enhancement of the CNS lesion (arrows) on T2-weighted post-gadolinium contrast axial images show the response to the treatment (for subfigure g the arrow indicates the location of relaspsed tumor). The positions pointed by the arrows are the locations of the tumor, and the other abnormalities on the images are due to the craniotomy. **(A)** No.1 patient: (a) on 2024/12/02 at relapse before the therapy of glofitamab combined with lenalidomide; (b) on 2025/02/15 after 2 cycles of the therapy; (c) on 2025/04/02 after 4 cycles of the therapy. **(B)** No.2 patient:(d) on 2024/10/19 at relapse before the therapy of glofitamab combined with lenalidomide; (e) on 2024/12/05 after 2 cycles of the therapy; (f) on 2025/01/25 after 4 cycles of the therapy. **(C)** No. 3 patient: (g) a PET-CT scan on 2024/12/02 at relapse before the therapy of glofitamab combined with lenalidomide; (h) on 2025/01/14 after 2 cycles of the therapy; (i) on 2025/03/07 after 4 cycles of the therapy. **(D)** No. 4 patient. (j) on 2025/03/28 at relapse before the therapy of glofitamab combined with lenalidomide; (k) on 2025/05/15 after 2 cycles of the therapy; (l) on 2025/06/23 after 4 cycles of the therapy.

The No.1 patient was a 54-year old man with PCNSL. His lymphoma lesions were located in the left paraventricular basal ganglia. After 4 cycles of RTM+BTKi (rituximab 375mg/m^2^ d1, thiotepa 30mg/m^2^ d2, methotrexate 3.5g/m^2^ d3, orelabrutinib 150mg qd) chemotherapy, he achieved CR. Then he received another 2 cycles of RTM+BTKi chemotherapy and 2 cycles of rituximab targeted therapy. However, 1 month later, he went to hospital again for blurred vision, and was diagnosed relapsed. For the first relapse of lymphoma, he received 1 cycle of chemotherapy with RTCS (rituximab 375mg/m^2^ d1, thiotepa 30mg/m^2^ d2, cytarabine 2g/m^2^ q12h d3~d4, selinexor 60mg qw), then a WBRT with 40Gy, then a high-dose thiotepa-based conditiong regimen, and then a successful ASCT. But unfortunately, in 2024-12, 3 months later after ASCT, he developed weak limbs and walking disorder, and was confirmed relapsed again. For the second relapse, as a variety of therapies had been used previously, he started to try a treatment with glofitamab combined with lenalidomide. Glofitamab was administered intravenously with step-up dose till up to 30mg, and lenalidomide was orally administered 15mg/day continuously. He achieved PR after 2 cycles of therapy of glofitamab combined with lenalidomide, although he still kept in PR in the following therapy, his tumor decreased and presented with no contrast enhancement by MRI, then he got very close to CRu after 4 cycles (**Figure 1**). During therapy, the patient was monitored carefully. Transient grade 1 CRS and ICANS once occurred after the first glofitamab administration, but no other adverse events (AE) were observed during the following treatment.

The No. 2 patient was a 58-year old woman with PCNSL. Her lymphoma lesions were located mainly in the right frontal lobe, corpus callosum, insular lobe, and partly in the right medial splenius capitis lymph node and bone marrow. She attained CR after 4 cycles of RTM+BTKi (rituximab 375mg/m^2^ d1, thiotepa 30mg/m^2^ d2, methotrexate 3g/m^2^ d3, orelabrutinib 150mg qd) chemotherapy. Then for subsequent treatment, she only agreed to receive orelabrutinib 150mg qd and refused other chemotherapy and WBRT. In 2024-10, 6 months later after the last chemotherapy, she presented with nausea, poor appetite, listlessness, and lethargy. Further examination confirmed relapse in the right posterior horn of the ventricle and the left cerebellum. At the time of recurrence, her physical condition with ECOG score >3 did not allow high-dose chemotherapy and ASCT. Then she received the therapy with glofitamab combined with lenalidomide. After 2 cycles of therapy she achieved CRu (**Figure 1**). She had grade 1 CRS and ICANS, as well as grade 2 myelosuppression in only the first and second cycles of glofitamab administration, but she recoverd soon after symptomatic treatment. By now she keeps in CR and is still in the 10^th^ cycle of the therapy with glofitamab combined with lenalidomide.

The No.3 patient was a 69-year old woman with PCNSL. Her lymphoma lesions were located in the left frontal lobe and basal ganglia. At first, She received 4 cycles of chemotherapy with RT+BTKi (rituximab 375mg/m^2^ d1, thiotepa 30mg/m^2^ d2, orelabrutinib 150mg qd), and then she achieved CR. But one day after that, she accidentally got a pulmonary tuberculosis which interupted the chemotherapy, and she started to be treated with only orelabrutinib. In 2024-12, 8 months after the interuption of chemotherapy, the PCNSL recurred near the right lateral ventricle of the centrum semiovale. In consideration of the patient’s advanced age, poor physical condition, and history of pulmonary tuberculosis, intensive chemotherapy and ASCT were not recommended, besides, she also refused WBRT. Then she received the therapy of glofitamab combined with lenalidomide. She got PR after 2 cycles of treatment, then got CRu after the 4^th^ cycle (**Figure 1**). She had a transient symptom of grade 1 CRS druing the first cycle of glofitamab administration. No ICANS or other AE was observed during subsequent treatment. Now she is still in the 8^th^ cycle of the therapy, and still in CRu.

The No.4 patient was a 51-year old woman with SCNSL. Eight years ago, she had a primary breast DLBCL, and received ASCT after 4 cycles of chemotherapy with R-CHOP combined with MTX. In 2024-05, she developed epilepsy and consciousness disturbance without any obvious cause. Imaging examination showed a tumor in the right frontal lobe of her brain. Then, a total body positron emission tomography-computed tomography (PET-CT) showed manifestations of only the intracranial tumor but no manifestations of tumor at other locations. Craniotomy and biopsy were performed and the result confirmed a DLBCL. Eight years ago, the next generation sequencing was applied to the breast tumor of the patient, and this time, the next generation sequencing was applied again to the intracranial tumor as well. The results are nearly the same. Therefore, a recurrence of previous DLBCL was considered. She then received 3 cycles of RTM+BTKi (rituximab 375mg/m^2^ d1, thiotepa 30mg/m^2^ d2, methotrexate 3.5g/m^2^ d3, orelabrutinib 150mg qd) chemotherapy and achieved CR. After that she received another 2 cycles of RTM+BTKi chemotherapy and then received ASCT again. But unfortunately, in 2025-03, 3 months after ASCT, she went to hospital complaining of headache and visual impairment, and lymphoma lesions were found recurred in her bilateral parieto-occipital lobe and the left splenium of her corpus callosum. At the second relapse, glofitamab plus lenalidomide were given for treatment. She achieved CR after 4 courses of treatment (**Figure 1**). During the first cycle of glofitamab, she once had fever, hyoxemia, and lethargy, which were recorded as grade 2 CRS and ICANS, Besides, she also had grade 1 myelosuppression. These symptoms were relieved after dexamethasone and symptomatic treatment. So far, she is still in CR and in the process of therapy with glofitamab combined with lenalidomide.

**Figure 2:**
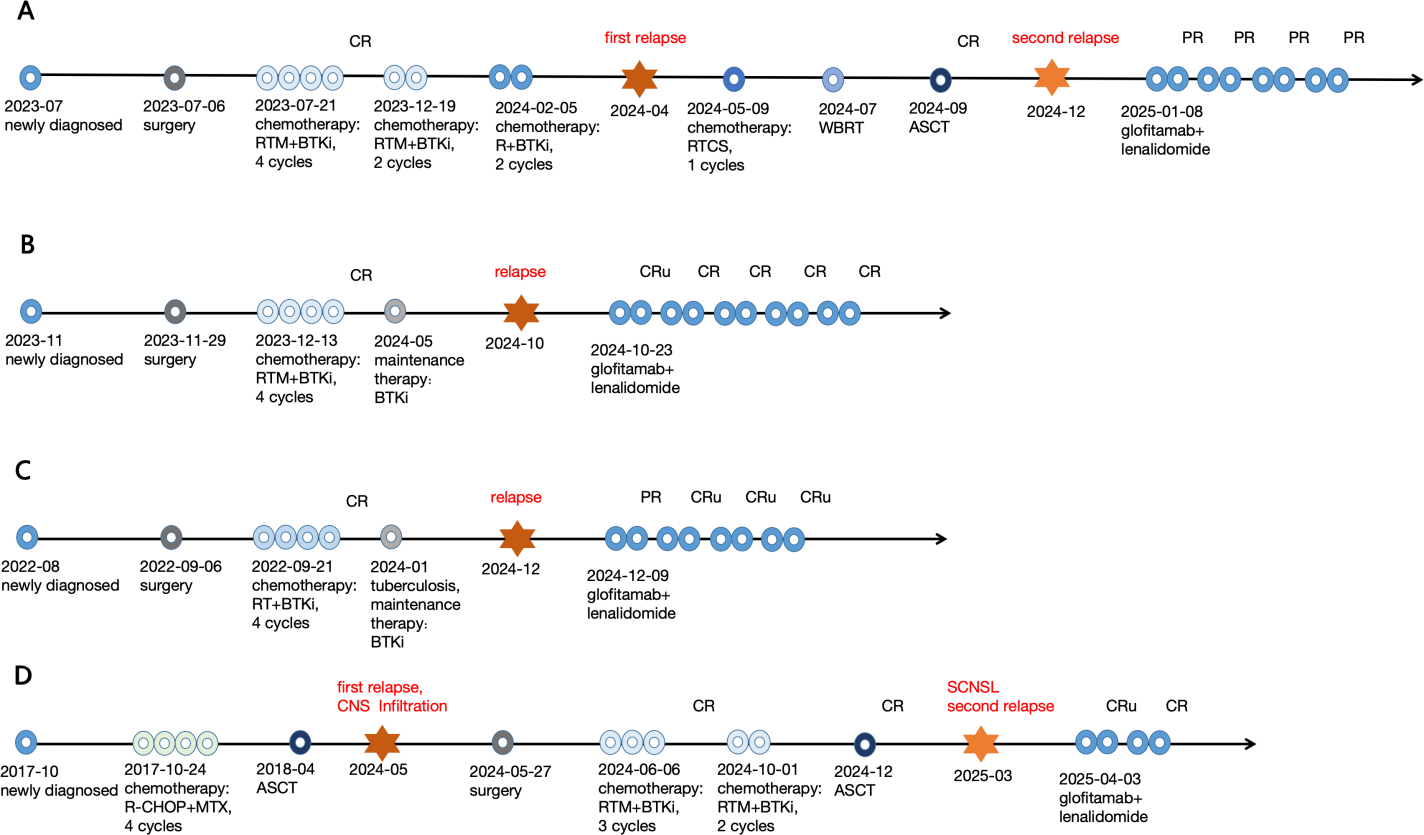
Therapy processes of the 4 R/R CNSL patients. **(A)** No.1 patient; **(B)** No.2 patient; **(C)** No.3 patient; **(D)** No.4 patient.

**Figure 2** illustrates the treatment processes of the 4 patients. The overall response rate (ORR) of them was 100% (4/4) after 2 cycles, with 2 patients in CRu and 2 patient in PR. After the 4^th^ cycle, they got deep remission (3 patients in CR/CRu, 1 in PR). The AE were mild and reversible. Only grade 1 or 2 CRS, ICANS, myelosuppression were recorded, no patient experienced CRS, ICANS and hematological toxicity of grade ≥3. No patient developed thrombosis or serious infection. Neither liver nor kidney dysfunction was observed, and no other unexpected AE, such as nausea or vomiting was observed. No treatment discontinuation occurred due to AE.

## 4 Discussion

CNSL is a rare sub-type of NHL, mainly of DLBCL. Treatment of CNSL is different from those of systemic lymphomas, mainly because of the blood-brain barrier, and it carries a worse prognosis when compared with DLBCL in other organ systems (30). CNSL’s first-line treatment consists of a high-dose MTX-based polychemotherapy, followed in eligible patients by a consolidation therapy with high-dose chemotherapy and subsequent ASCT (31). Despite recent improvements in the front-line treatment, up to 60% of patients eventually relapse (32), moreover, about 25% of patients fail to respond to initial treatment (33). So the prognosis of R/R CNSL is grim, and the optimal treatment is poorly elucidated as there have only been a limited number of studies conducted in this setting (33).

Historically, WBRT was used for newly diagnosed CNSL, but this led to frequent relapses with a poor median OS of only 12 to 18 months (8). The use of radiation in the induction phase of CNSL is decreasing with time, as studies have shown that WBRT in combination with MTX produces significant neurotoxicity and is not associated with an increased OS, although it does increase progression-free survival (PFS) (34).

ASCT is a promising consolidative strategy, especially for young healthy patients with CNSL (35). But for R/R CNSL patients, most patients are unable to undergo ASCT due to age, drug toxicity, intolerance to high dose chemotherapy, poor efficacy of the induction therapy, failure of stem cell mobilization, et al. Toxicity, including transplantation-related mortality, is also an important issue in ASCT. Even if ASCT is performed, the efficacy cannot be guaranteed upfront for R/R CNSL patients. Moreover, some R/R CNSL patients have already received ASCT in the past. So the effective treatment options for R/R CNSL patients are limited. Novel therapeutic agents with excellent CNS penetration, better efficacy, and tolerable toxicity profile are urgently needed. Given these limited options for salvage therapy in CNSL, the development of novel therapies based on molecular insights from tumor profiling has led to additional targeted options either recently approved or currently under investigation for R/R disease (36).

Many novel small molecule drugs, such as BTKi (15), PD-1 inhibitor (16), PI3K inhibitor (17), XPO-1 inhibitor (18), immunomodulatory drug (19–22) have been used to the R/R CNSL. Although they are found to be active in R/R CNSL patients, the responses are usually short, and it is necessary to explore more options of combination therapies.

CAR-T therapy is a promising immunotherapy. It was approved by the United States Food and Drug Administration (FDA) for R/R DLBCL in 2017. As DLBCL is the main pathological type of CNSL, CAR-T therapy is expected to be beneficial (37). But in fact, in early years, patients of CNSL were excluded from the CAR-T therapy of many clinical trials. However, subsequent studies with real-world experience indicated CAR-T therapy would be an effective treatment for CNSL patients with a manageable safety profile (38). Although it is proved that, the efficacy of CAR-T therapy for CNSL had an acceptable safety profile, the remission does not last long, the median PFS is only 3 months and the relapse rate is 80% (39). Besides, there are not many reports of CNSL patients who were treated with CAR-T therapy, and most patients cannot afford the high price. Thus, the efficacy and safety of CAR-T therapy in CNSL remain theoretically effective but unknown.

Glofitamab is a CD20×CD3 bispecific antibody that is promising for treating R/R DLBCL (40). Glofitamab has a novel 2:1 tumor-T-cell binding configuration that confers bivalency for CD20 (B cells) and monovalency for CD3 (T cells), leading to the engagement and redirection of patients’ existing T cells to eliminate malignant B cells (41).

Previously in a study (41), 155 R/R DLBCL patients received at least two lines of therapies, at a median follow-up of 12.6 months. Among the 155 patients who received the phase 2 dose of glofitamab, 39% of the patients got CR. The median time to CR was 42 days, 78% of the patients still kept in CR at 12 months. The 12-month PFS was 37% (41). Many studies (41–43) had confirmed that glofitamab therapy was effective for R/R DLBCL.

Recently, Godfrey JK, et al (44), reported 4 R/R SCNSL patients who were all treated with the CD20×CD3 bispecific antibody, glofitamab. Among the 4 R/R SCNSL, 3 patients showed objective radiological and clinical improvement after glofitamab treatment, and treatment related CRS was only grade 1~2, and no ICANS occurred. This report confirmed that glofitamab could stimulate immune-cell infiltration of CNSL, and induce responses in CNSL (44). Wang W, et al (45), reported one relapsed PCNSL patient treated with glofitamab therapy. The patient achieved CR after two cycles of treatment with glofitamab, and continued to experience remission and maintain an optimistic survival status, only grade 1 CRS reaction occurred in the first cycle of treatment (45).

Glofitamab can provide a durable response in R/R DLBCL patients even in a heavily treated and highly refractory DLBCL, and the glofitamab is now approved for the treatment of R/R DLBCL. Glofitamab may be limited to CNSL previously as it couldn’t fully cross the blood-brain barrier. The recent literatures report (44, 45) that glofitamab is effective in both SCNSL and PCNSL. Although the concentration of glofitamab in CSF is only 0.1~0.4% of that in the peripheral blood (44), the low-level of glofitamab in the CSF has the capability of eliciting responses in CNSL. The low-level of glofitamab in the CSF can induce T cell activation and contribute significantly to the clearance of lymphoma from the CSF (44). These data therefore support future prospective investigation of glofitamab in clinical trials of CNSL to meet the need of this patient population.

Lenalidomide is a second-generation immunomodulatory drug with pleiotropic antitumor effects including stimulation of natural killer and T-cell expansion (19). Lenalidomide monotherapy or combination therapy can improve the efficacy of treatment to R/R ABC or non-GCB DLBCL (46), newly diagnosed high-risk DLBCL with MYC gene rearrangement (47), and DLBCL in immune-privileged sites (19–22). Lenalidomide can successfully penetrate the blood-brain barrier as small molecules (48). Pathologically, most CNSL are DLBCL and are characterized as the ABC or non-GCB subtype, so patients with CNSL may benefit from lenalidomide. A phase 1 study evaluated the efficacy of lenalidomide in 14 R/R CNSL patients (19). It was found that the single-agent response rate of lenalidomide was 64.3%(9/14), 6 of the 14 patients maintained response ≥9 months, and 4 patients maintained response for more than 18 months. Therefore, CNSL patients can indeed benefit from lenalidomide. A prospective single-arm phase II study of lenalidomide combined with pemetrexed for salvage treatment of R/R PCNSL showed that the ORR was 68.4%, the median PFS and OS were 6 and 18 months, respectively (20). Another prospective phase II study of lenalidomide in combination with rituximab in R/R PCNSL, showed the best ORR was 67% (CR/CRu 40%, PR 27%), and the median PFS and OS were 7.8 months and 17.7 months (49). So lenalidomide had proved to be a promising therapeutic method for R/R CNSL. Compared with lenalidomide monotherapy, multidrug combination therapy is recommended.

Immunomodulatory drugs have been reported to enhance the cytotoxicity and cytokine production of T cells, suggesting that immunomodulatory drugs may work synergistically with T-cell engager therapies such as CD3-bispecific antibodies (50). A previous study had demonstrated that the immunomodulatory drugs substantially enhanced tumor cell killing that was induced by CD3 bispecifics, and increased the CD8+ T-cell proliferation and expansion (50). So combination of lenalidomide and CD3-bispecific antibody glofitamab is probably to have a synergistic effect on CNSL. Lenalidomide is usually applied in hematological malignancy at a dose of 25 mg/day, but perhaps a lower dose may be more tolerable to patients, and may increase the administration time and the efficacy (51). So, in our case, we added continuous lenalidomide 15mg/day to be combined with glofitamab in R/R CNSL in order to enhance the therapeutic effect.

In this report the 4 R/R CNSL patients included 3 with PCNSL, and 1 with SCNSL. The 4 R/R CNSL patients were treated with glofitamab combined with lenalidomide. After 2 cycles of treatment, all patients achieved rapid remission (2 in CRu and 2 in PR), and they got deep remission after 4 cycles (3 in CR/CRu, 1 in PR). To our knowledge, this is the first report of R/R CNSL achieving remission through glofitamab combined with lenalidomide. During the whole immunochemotherapy phase, their AE were mild and reversible, they experienced neither CRS, ICANS nor hematological toxicity of grade 3 or above. Neither liver nor kidney dysfunction was observed, and no other AE were observed. No treatment discontinuation occurred due to AE. Results show the glofitamab combined with lenalidomide is a charming therapy for R/R CNSL.

## 5 Conclusions

Although CNSL is highly responsive to chemotherapy and radiation therapy in the first-line setting, the recurrence rate remains unacceptably high and prognosis remains extremely poor. There is still a lack of standard salvage treatment for R/R CNSL, and clinical trials should still be considered as preferred options. New treatment methods with novel measures different from conventional therapies are of great interest. The glofitamab-lenalidomide combination shows promising activity and excellent tolerability in R/R CNSL, potentially addressing the critical need for effective salvage regimens. However, to further verify the efficacy and security of glofitamab combined with lenalidomide treatment in R/R CNSL, more case studies and prospective clinical trials are still required.

## Data Availability

The original contributions presented in the study are included in the article/supplementary material. Further inquiries can be directed to the corresponding author.

## Glossary

CNSL: central nervous system lymphoma
NHL: non-Hodgkin’s lymphoma
PCNSL: primary central nervous system lymphoma
SCNSL: secondary central nervous system lymphoma
CNS: central nervous system
WHO: the World Health Organization
DLBCL: diffuse large B-cell lymphoma
ABC: activated B-cell-like
non-GCB: non-germinal center B-cell-like
MTX: methotrexate
R/R: relapsed/refractory
OS: overall survival
ASCT: autologous stem cell transplantation
WBRT: whole-brain radiotherapy
CAR-T: chimeric antigen receptor T-cell
CRS: cytokine-release syndrome
ICANS: immune effector cell-associated neurotoxicity syndrome
BTKi: Bruton tyrosine kinase inhibitor
PD-1: programmed cell death protein 1
PI3K: phosphoinositide 3-kinase
XPO-1: exportin 1
ECOG: Eastern Cooperative Oncology Group
G-CSF: granulocyte colony-stimulating factor
MRI: magnetic resonance imaging
CSF: cerebrospinal fluid
CR: complete response
CRu: unconfirmed complete response
PR: partial response
SD: stable disease
PD: progressive disease
PRO-CTCAE: Patient-Reported Outcomes version of the Common Terminology Criteria for Adverse Events
ASTCT: the American Society for Transplantation and Cellular Therapy
AE: adverse events
PET-CT: total body positron emission tomography-computed tomography
ORR: overall response rate
PFS: progression-free survival
FDA: Food and Drug Administration.
